# Temporal and Spatial Variation of the Skin-Associated Bacteria from Healthy Participants and Atopic Dermatitis Patients

**DOI:** 10.1128/msphere.00917-21

**Published:** 2022-02-23

**Authors:** Christopher J. Barnes, Maja-Lisa Clausen, Maria Asplund, Linett Rasmussen, Caroline Meyer Olesen, Yasemin Topal Yüsel, Paal Skytt Andersen, Thomas Litman, Anders Johannes Hansen, Tove Agner

**Affiliations:** a The Globe Institute, Faculty of Health, University of Copenhagen, Copenhagen, Denmark; b Department of Dermatology, Bispebjerg Hospital, University of Copenhagen, Copenhagen, Denmark; c Department of Bacteria, Parasites and Fungi, Statens Serum Insitute, Copenhagen, Denmark; d Department of Immunology and Microbiology, LEO Foundation Skin Immunology Research Center, University of Copenhagen, Copenhagen, Denmark; University of Wisconsin-Madison

**Keywords:** Atopic dermatitis, eczema, metabarcoding, *Staphylococcus aureus*, skin microbiome, spatial-temporal variation, bacteria, atopic dermatitis, temporal variation

## Abstract

Several factors have been shown to influence the composition of the bacterial communities inhabiting healthy skin, with variation between different individuals, differing skin depths, and body locations (spatial-temporal variation). Atopic dermatitis (AD) is a chronic skin disease also affecting the skin-associated bacterial communities. While the effects of AD have been studied on these processes individually, few have considered how AD disrupts the spatial-temporal variation of the skin bacteria as a whole (i.e., considered these processes simultaneously). Here, we characterized the skin-associated bacterial communities of healthy volunteers and lesional and nonlesional skin of AD patients by metabarcoding the universal V3-V4 16S rRNA region from tape strip skin samples. We quantified the spatial-temporal variation (interindividual variation, differing skin depths, multiple time points) of the skin-associated bacteria within healthy controls and AD patients, including the relative change induced by AD in each. Interindividual variation correlated with the bacterial community far more strongly than any other factors followed by skin depth and then AD status. There was no significant temporal variation found within either AD patients or healthy controls. The bacterial community was found to vary markedly according to AD severity, and between patients without and with filaggrin mutations. Therefore, future studies may benefit from sampling subsurface epidermal communities and considering AD severity and the host genome in understanding the role of the skin bacterial community within AD pathogenesis rather than considering AD as a presence-absence disorder.

**IMPORTANCE** The bacteria associated with human skin may influence skin barrier function and the immune response. Previous studies have attempted to understand the factors that regulate the skin bacteria, characterizing the spatial-temporal variation of the skin bacteria within unaffected skin. Here, we quantified the effect of AD on the skin bacteria on multiple spatial-temporal factors simultaneously. Although significant community variation between healthy controls and AD patients was observed, the effects of AD on the overall bacterial community were relatively low compared to other measured factors. Results here suggest that changes in specific taxa rather than wholesale changes in the skin bacteria are associated with mild to moderate AD. Further studies would benefit from incorporating the complexity of AD into models to better understand the condition, including AD severity and the host genome, alongside microbial composition.

## INTRODUCTION

The skin is the largest organ of the human body, serving as a protective barrier against the external environment and allowing the body to retain water (1). It is colonized by millions of bacteria, fungi, and viruses – collectively called the skin microbiome (2). A healthy skin microbiome contains many thousands of commensal bacterial species, where they serve as the skin’s first line of defense, preventing the colonization of pathogenic bacterial species (3) as well as helping modulate host immune defense (4). Pathogenic microbes can disrupt commensal communities associated with the skin, with for example Staphylococcus aureus and Streptococcus pyogenes causing skin infections (5). Common skin disease, such as atopic dermatitis (AD), is associated with the colonization of S. aureus in over 75% of cases. Other skin disorders, such as acne vulgaris, psoriasis, and hidradenitis suppurativa, have also been associated with complex changes in the microbiome (6, 7). However, it is not known whether changes in the microbiome are a symptom or a cause of disease.

AD affects up to 20% of children and 5 to 7% of adults worldwide (8, 9). It is an intensely pruritic inflammatory skin disease, giving rise to decreased quality of life. AD is characterized by a dysregulated immune response and an impaired skin barrier. The bacterial communities of nonlesional and lesional skin from AD patients have been observed to differ from healthy control skin and each other (10), but, generally, the bacterial communities of adult AD patients have reduced bacterial richness and increased Staphylococcus aureus abundance (11). However, it is not known whether the altered bacterial community drives AD or is opportunistically colonized by S. aureus due to a disrupted barrier.

It is vital to have a thorough understanding of the healthy microbiome to fully comprehend the role of the microbiome within AD pathogenesis. However, microbial communities are enormously complex and variable. With the advent of improved DNA sequencing technologies, the bacterial communities from hundreds of skin samples can be profiled at once, revealing the taxonomy, although often not to species level, and relative abundances of a substantial proportion of the bacterial diversity within (12). In the preceding decade, many studies have aimed to understand the variation of skin bacteria (13). Consequently, it has been shown that the skin bacterial communities of healthy individuals vary between individuals (14), between different locations on the body, particularly between different environments, such as sebaceous, moist, and dry skin environments (15, 16), and in depths between the different skin layers (3, 17, 18). Despite considerable variation in the environmental bacteria that surrounds individuals (19), the skin communities are relatively stable over time for most people across skin habitats (20). While many studies have looked at understanding one or a few of these temporal and spatial parameters individually, few have attempted to determine the relative effects of these parameters simultaneously. These parameters may exert confounding effects that go unmeasured when single considered parameters are considered in isolation, such as AD status (21). Consequently, the relative effect of AD on the spatial-temporal variation of the skin microbiome remains understudied and thus the cumulative effects of AD on the skin bacterial community are much less understood.

**FIG 1:**
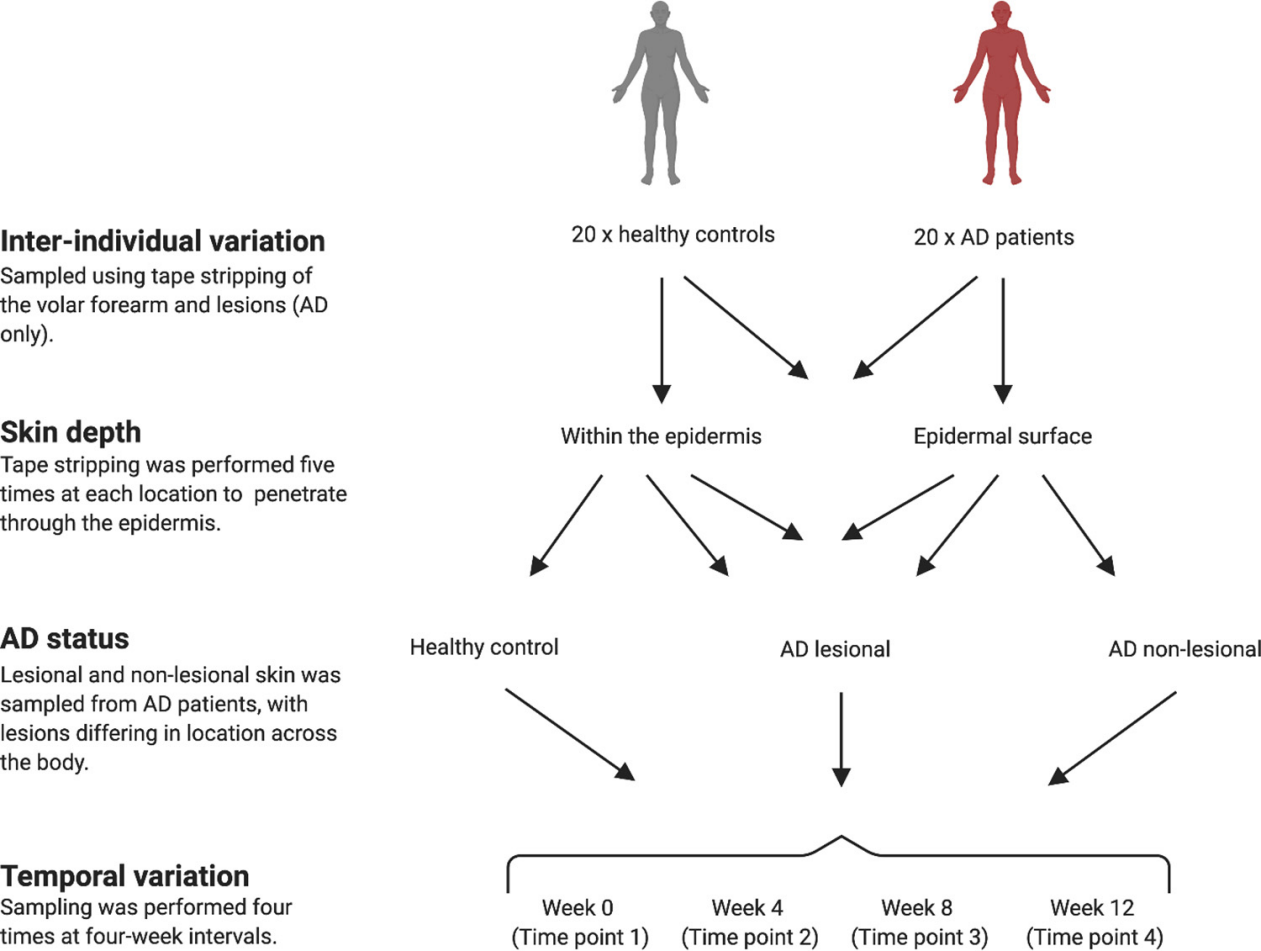
Schematic of sampling. Sampling was performed on 20 AD patients and 20 healthy controls, which was repeated four times at 4-week intervals. Sampling was performed using tape stripping, with five tape strips performed at each location, and tape 1 (considered the epidermal surface) and tape 5 (considered within the epidermis) were analyzed.

In this study, we compared the relative variation of spatial (inter-individual variation, skin differing skin depths) and temporal (repeated sampling) in AD patients and healthy controls. The bacterial communities of adult AD patients (lesional and nonlesional skin) and healthy controls were sampled a total of 4 times at 4-week intervals over 12 weeks. Bacterial samples were collected by tape stripping the skin of the volar forearm 5 times (and lesional skin of AD patients), thereby sampling the surface of the epidermis (tape 1) and within the epidermis (tape 5) (22) (Fig. 1; complete metadata listed in Data Set S1 available at https://sid.erda.dk/sharelink/hkdDwYKYmc). The bacterial communities were characterized by DNA metabarcoding of the universal bacterial 16S rRNA V3-V4 region (Data Set S1). We subsequently attempted to quantify the relative effect sizes of inter-individual variation, skin depth (epidermis surface, within the epidermis), and temporal variation with the bacterial-associated skin communities associated with healthy skin and nonlesional skin of AD patients. We further explored the effects of AD severity (assessed as objective scoring of AD [O-SCORAD]) (23) and filaggrin mutations on the bacterial community of AD patients. Finally, we identified a core community within participants comprising of amplicon sequence variants (ASVs) that were stable over space and time within participants, before identifying whether AD significantly impacts upon the composition or preponderance of the core skin bacteria.

## RESULTS

### Overall composition and regulation of the skin-associated bacterial communities.

**FIG 2:**
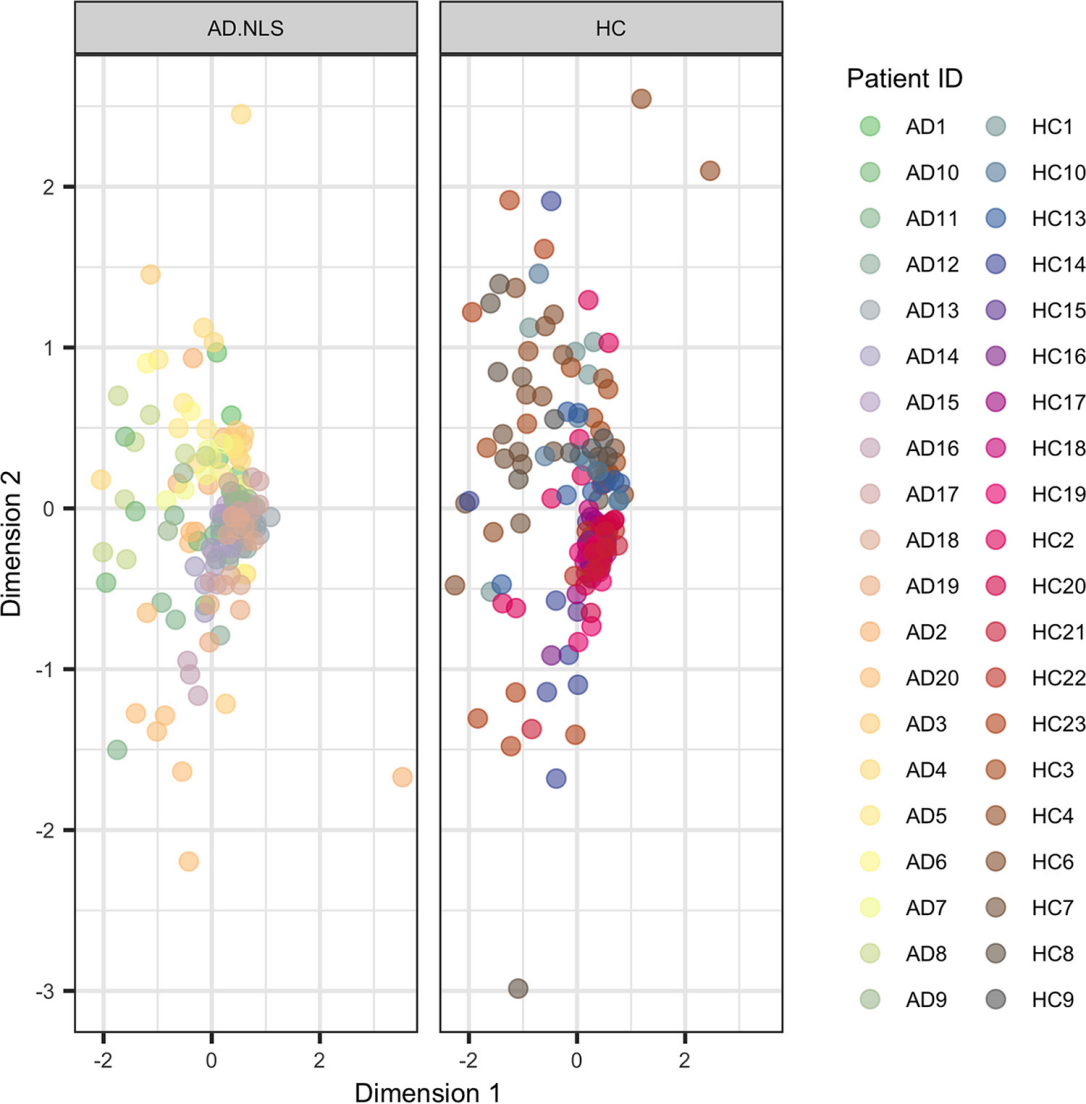
Nonmetric multidimensional scaling was performed to visualize the skin-associated bacterial communities (produced by metabarcoding the 16S rRNA region with universal bacterial primers). Color represents individual patients and facets are divided by AD status (from AD patients: AD.LS, lesional; AD.NLS, nonlesional; HC, healthy control), revealing significant similarity between samples from the same patient. All four time points and skin depths are included under each patient as individual data points.

Initially, all samples from nonlesional and healthy skin were used to correlate inter-individual variation (patientID), skin depth (epidermal surface or within the epidermis), temporal variation (the four sampling time points), and AD status (nonlesional and healthy control) against bacterial community variation using PERMANOVA (Table 1). Interindividual variation was by far the biggest parameter that affected the community composition (R^2^ of 0.307), accounting for 30.7% of community variation, as demonstrated by clustering by similarity within in the nMDS plot (Fig. 2). However, there were small but significant effects of skin depth (2.0%) and AD status (0.9%) of community variation. However, no significant temporal variation was found.

**TABLE 1:**
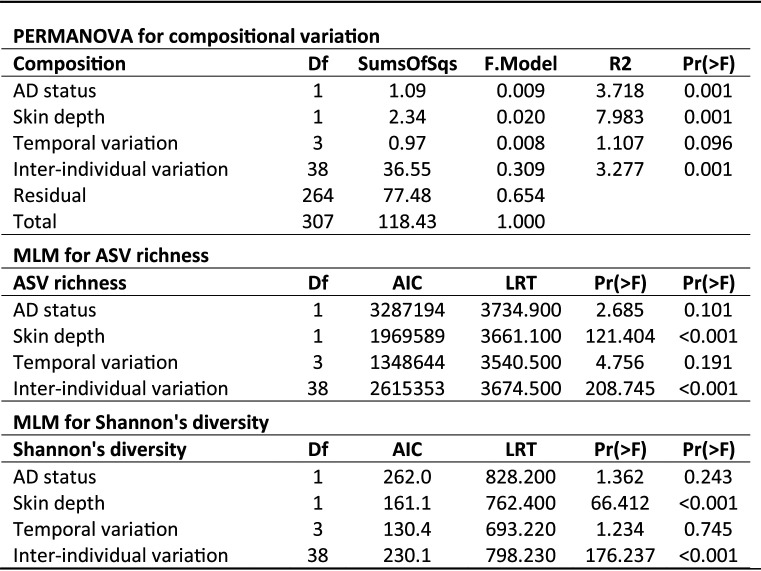
Significant differences of the skin bacterial community composition (with PERMANOVA), ASV richness, and Shannon's diversity (both using generalized linear modeling) were assessed against AD status, skin depth, temporal variation, and interindividual variation

**FIG 3:**
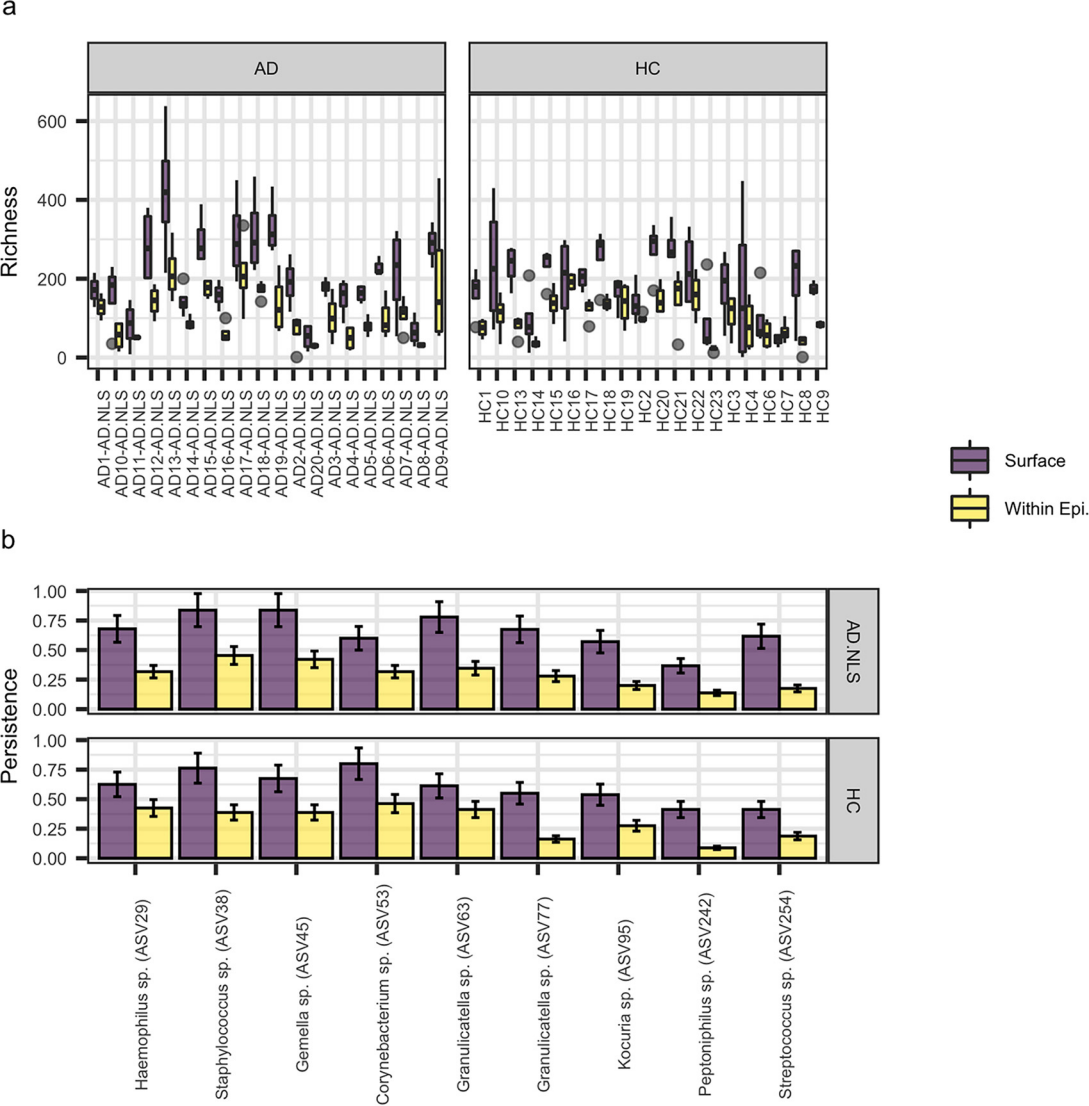
(A) Boxplot demonstrating the significant variation in ASV richness between individuals and the significant decline associated with sampling at the epidermal surface to within the epidermis (from tape 1 to tape 5). Error bars represent the standard error of the mean (SEM) with replicates coming from the aggregating data from each time point. (B) The within-patient persistence of each ASV was compared between the epidermal surface and within the epidermis using paired *t* tests (partitioned into skin condition for visualization only). A total of 322 ASVs significantly varied between skin layers (*Q* value < 0.05), and the nine most significant were plotted (*Q* value < 0.0001). Error bars represent the standard error of the mean (SEM).

Additionally, α-diversity measures (ASV richness and Shannon’s diversity) were correlated against these parameters using generalized linear modeling (GLM). Both interindividual variation (Table S2) and skin depth (Fig. 3A) varied significantly in both metrics, with significantly higher ASV richness at the epidermal surface than within the epidermis (Table 1). Meanwhile, there were no significant differences in ASV richness and Shannon’s diversity associated with AD status and temporal variation.

Similar to many previous studies of the skin-associated bacteria, communities were dominated by the Proteobacteria (11.6%, 46.5 ASVs), Firmicutes (29.2%, 43.7 ASVs), Actinobacteria (23.0%, 35.3 ASVs) in mean ASV richness and relative abundance. However, there were substantial deviations from this pattern within both patients and volunteers. For example, one healthy volunteer consistently had a relative abundance of *Anaerococcus* that was orders of magnitude higher than all other participants. The Corynebacterium was the most abundant genus in both relative abundance and ASV richness (10.6% and 14.2 ASVs), followed by Acinetobacter (6.2% and 8.8 ASVs). The Staphylococcus genus was the third most abundant genus in both relative abundance (6.0%) and ASV richness (7.0 ASVs) across the nonlesional and healthy controls (16) and was significantly higher in relative abundance (*H *= 8.27, *P* = 0.004) but not ASV richness (*H *= 3.76, *P* = 0.052) in the nonlesional samples (5.4%, 7.2 ASVs) compared to the healthy controls (4.6%, 6.0 ASVs).

### Interindividual variation of the skin-associated bacterial communities.

Inter-individual variation was the primary source of variation in the richness and composition of the skin-associated bacterial communities. In addition to α-diversity (ASV richness and Shannon’s diversity), differences in β-diversity (i.e., the level of variation between samples) were also explored as the difference in ASV richness between samples, community membership, or community similarity. Significant β-diversity was initially assessed between all individuals (i.e., both AD patients and healthy controls), and significantly varied in all three metrics (using Kruskal-Wallis tests; Table S2; Fig. S1), with the rate of change of bacterial communities found to be highly personalized. We further explored whether there were significant differences in β-diversity between AD patients and healthy controls (i.e., were AD patients bacterial communities more variable over time), but there were no significant differences in any of the three β-diversity metrics (Table S3; Fig. S2).

Given this overwhelming impact of interindividual variation on the community composition and ASV richness of the skin-associated bacteria, we accounted for interindividual variation in all subsequent statistics.

### Skin depth effects on the bacterial community.

Skin depth was the second most important factor correlating with the skin bacterial community composition and ASV richness (Table 1), with nearly twice as many ASVs at the epidermal surface (197.9 ASVs) that within the epidermis (106.8 ASVs; with nonlesional and healthy controls considered together). Similarly, Shannon’s diversity significantly declined from 5.35 to 4.63 between the two (Table 1; Fig. 3A). Despite the reduced ASV richness within the epidermis, there were no significant differences in β-diversity between the different skin depths (i.e., samples were as variable as each other within each subject over time; Table S4).

We further investigated the dynamics of individual bacteria between skin depths by performing paired *t* tests on the within-participant persistence of all common ASVs occurring in more than 5 samples. A total of 322 ASVs (*Q* < 0.05) were identified that significantly varied between skin depths (Fig. 3B and Table S5). Some of the most common ASVs strikingly decreased in persistence between skin layers. This included a *Granulicatella* species (ASV77) that dropped from persistence of 0.61 at the epidermal surface to persistence of 0.22 within the epidermis, a Staphylococcus species (ASV38) that dropped from 0.80 to 0.42, and a *Gemella* species (ASV45) that dropped from 0.76 to 0.40. Meanwhile, only a single ASV was found more commonly within the epidermis than at the surface, an uncommon *Gracilibacteria* species (ASV783) that went from within-person persistence of 0.01 (1%) at the epidermal surface to 0.02 (2%) within the epidermis. Additionally, many ASVs were disproportionately found within the samples from the epidermal surface than within the epidermis or almost absent from within the epidermis (Fig. S3). These tended to be low abundance ASVs with low within-participant persistence, which could likely be environmental contaminants to the skin.

The effect of AD status (HC [N = 188] and NLS [N = 178]) was relatively small (0.92%) in the initial community analyses (Table 1). We, therefore, explored whether the AD effects are better observed by partitioning the communities into to epidermal surface (tape 1 samples; N = 262) and within the epidermis (tape 5 samples; N = 256) and reanalyzing for AD status effects on the communities independently. There were slight increases in the percentages of community variation explained by partitioning communities at different skin depths, explaining 1.4% at the epidermal surface and 1.1% within the epidermis (Table 2). However, neither ASV richness nor Shannon’s diversity declined against healthy controls at either skin depth (Table 2). Similarly, β-diversity was compared between the AD patients and healthy controls at both skin depths separately to determine whether AD samples varied more across time when the confounding effect of sampling different skin layers was accounted for. However, there were no significant differences observed (Table 2).

**TABLE 2:**
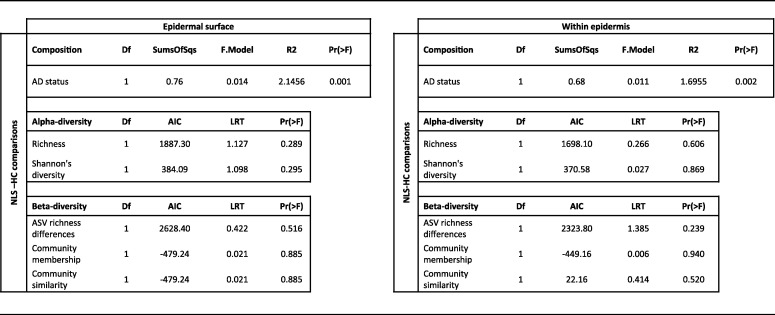
Significant variations associated with AD status either nonlesional skin from AD patients or healthy controls were assessed using mixed linear modeling. α-diversity (ASV richness and Shannon’s diversity) and β-diversity (ASV differences, community membership, and community similarity) were assessed using mixed linear modeling and PERMANOVA for community composition while accounting for significant interindividual variation with patientID as the random effect or strata)

**FIG 4:**
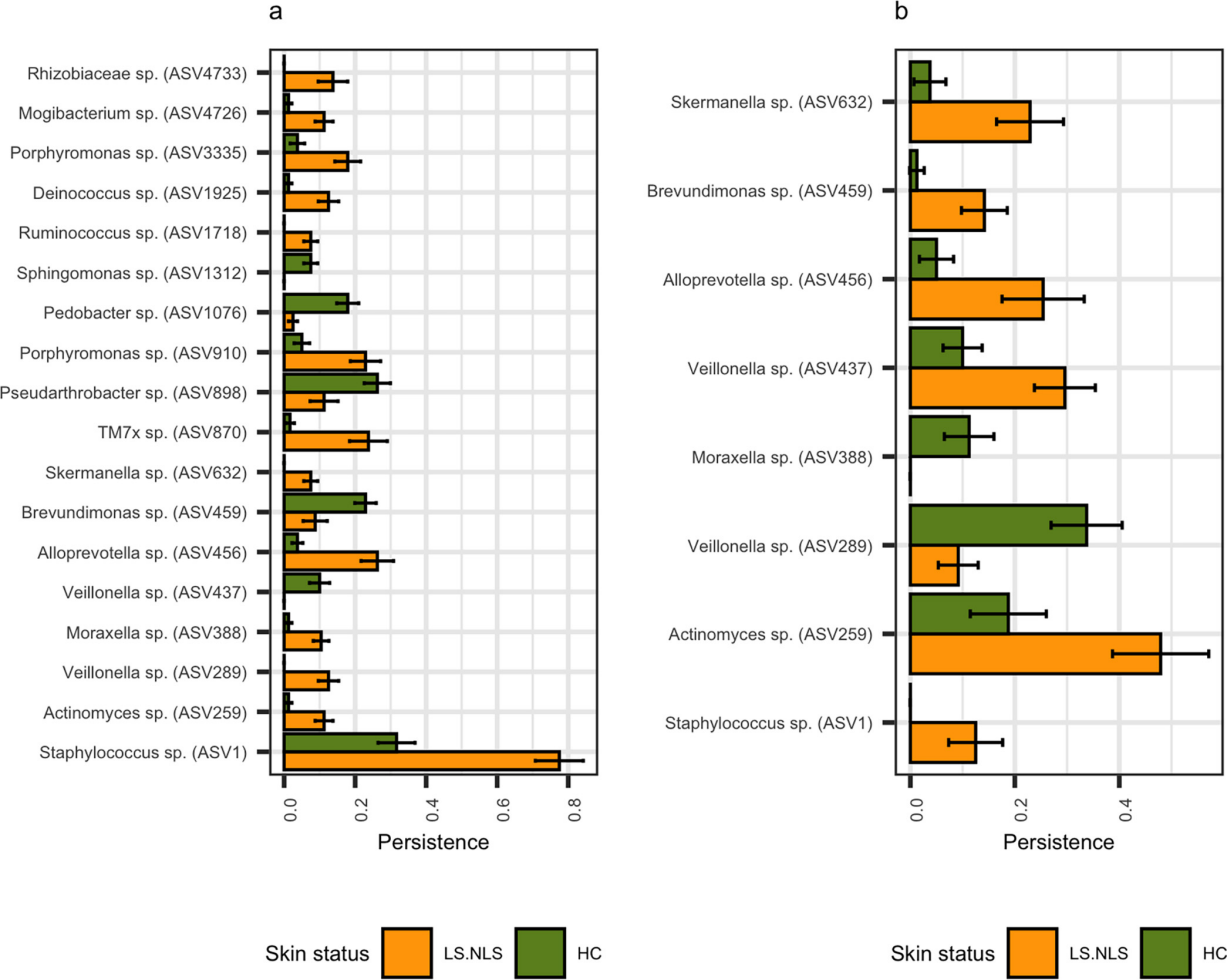
Within-patient persistences were calculated for individual ASVs and analyzed for significant differences between AD statuses (nonlesional skin of AD patients and healthy controls) using mixed linear modeling (patientID as a random effect). Samples were partitioned by skin depth, analyzed at the epidermal surface (A) and within the epidermis (B), with 61 and 29 ASVs found to significantly vary, respectively (*P* < 0.05), and the most significant are plotted (*P* < 0.001). It should be noted that no ASVs significantly varied after multiple corrections (*Q* < 0.05). Error bars represent the standard error of the mean (SEM).

Finally, mixed linear modeling was performed to identify the specific ASVs associated with AD status, using their within-participant persistence in healthy controls and nonlesional skin, where skin depths were considered separately. At the surface, 1124 ASVs were tested. ASVs were included if there were non-zero persistence in more than 5 patients. It was found that 61 ASVs significantly varied between AD statuses (Table S6; *P* < 0.05). Within the epidermis, only 590 ASVs were tested and 29 significantly varied between AD statuses (Table S6). At both levels, a Staphylococcus species (ASV1) was the most persistent ASV and was significantly more persistent within AD patients than in healthy controls. There was also a Corynebacterium species (ASV235) that was significantly more persistent within AD patients than healthy controls at both skin depths. It should be noted, however, that despite substantial differences within-participant persistence being observable (Fig. 4), none were found after correction for multiple comparisons (*Q *< 0.05).

### The bacterial community within nonlesional samples significantly varied with AD severity and filaggrin mutations.

The bacteria of the nonlesional AD communities were further explored with AD severity (O-SCORAD) and host filaggrin mutations (with the two skin layers analyzed separately). O-SCORAD varied from mild to moderate AD within patients and was correlated with 1.7% of community variation at the epidermal surface but not within the epidermis (Fig. S4A; Table S7). However, there were no significant differences in α- or β-diversity associated with changing O-SCORAD at either skin depth.

Filaggrin mutations were found in 10 out of 20 AD patients sampled, and the presence of these mutations correlated with 2.7% of community variation at the epidermal surface and 1.8% within the epidermis. There were no significant differences in α- or β-diversity at either skin depth (Fig. S5A and Table S8).

### The lesional bacterial communities differed from healthy controls but not the nonlesional communities.

As part of sampling AD patients, lesional skin was also sampled from patients at all 4 time points at both the epidermal surface and within the epidermis. However, lesional skin was not always present on patients adjacent to the main volar forearm-sampling site. Therefore, lesional skin was sampled at differing body locations, which also differed in some patients over time (volar forearm [N = 55], back [N = 14], elbow crease [N = 40], inguinal [N = 7], leg [10], neck [N = 10], wrist [N = 16]). There was a pronounced effect of the lesional skin location at the epidermal surface (14.5%) and within the epidermis (12.7%), while Shannon’s diversity also significantly varied between sampling locations at the epidermal surface (Fig. S6 and Table S9), which was highest in the volar forearm, elbow crease, and leg and the lowest in the inguinal.

The lesional communities were compared to the healthy controls and nonlesional samples (skin layers separately). When the lesional samples were compared to nonlesional communities, there were also no significant differences in community composition or α-diversity (Table S10). There were also no significant differences in α-diversity between the lesional and healthy controls (Table S10). However, the lesional communities were significantly different from the healthy controls at both skin layers, accounting for 1.3% at the epidermal surface and 1.2% within the epidermis.

As within the nonlesional communities, the effects of AD severity (as O-SCORAD) and filaggrin mutations were explored on the lesional communities. O-SCORAD correlated with the community composition of the bacterial community (1.7%), but only at the epidermal surface (Fig. S4B), while there was no correlation between α or β-diversity community and O-SCORAD (Table S7). Filaggrin mutations correlated with 2.1% of community variation at the epidermal surface and 2.0% within the epidermis (Fig. S5B and Table S8). Similarly, there were no correlations with α-diversity at either skin depth.

### Identification of a core bacterial community.

A core community (>0.7 within-patient persistence) was identified for each participant (samples from temporal replicates and skin depths pooled, but lesional samples were excluded from AD patients). In total, 355 ASVs were part of one or more patients’ core bacterial community. However, of these, 90 ASVs were in 0.7 or more in persistence across all samples (i.e., detectable in 70% or more of samples), and were considered ‘ubiquitous’ ASVs.

The ASVs of the core community accounted for a mean of 28.7% of total relative abundance for the nonlesional samples, and 23.0% for the healthy controls, but was not significantly different between the two (*H *= 0.73, *P* = 0.394). The core community accounted for a mean of 33.9 ASVs in the nonlesional community and 26.8 ASVs in the healthy controls. Similarly, there was no significant difference in the ASV richness of the core community between AD statuses (*H *= 3.77, *P* = 0.394). Additionally, a PERMANOVA revealed no significant difference in the composition of the core communities between AD patients and healthy controls (R^2^ = 0.027, *P* = 0.349).

## DISCUSSION

**FIG 5:**
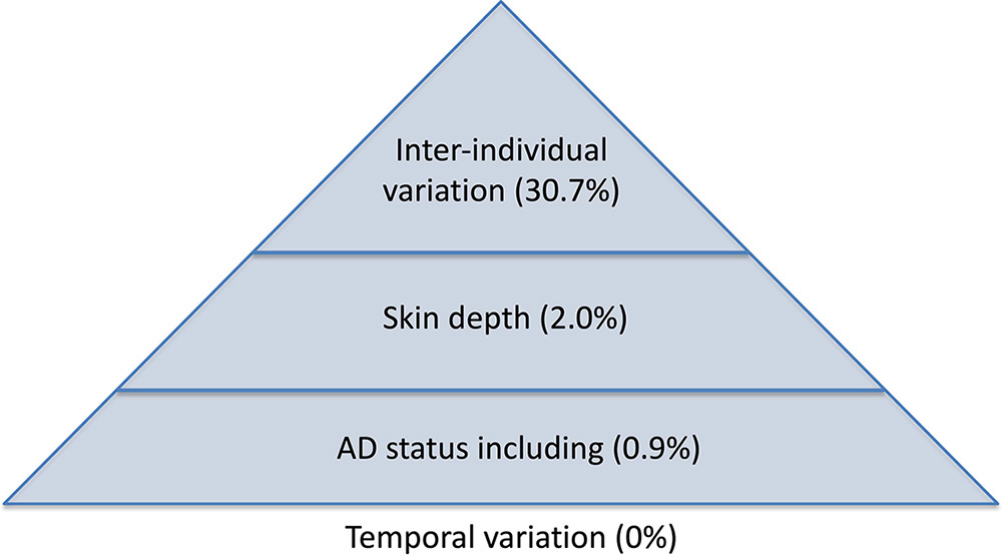
Hierarchy of factors correlating with the bacterial communities, with interindividual variation correlating with the most community variation, followed by skin depth, then small effects of skin condition. There was no significant temporal variation found.

Inter-individual variation had by far the largest effect on the bacterial communities of the skin. Skin depth had the second-largest effect, followed by AD status. Temporal variation was observed to be nominal within our study. Lesional and nonlesional communities did not significantly differ from each other, but both differed from healthy controls (Table S10). The relative effect sizes equated to roughly a 25:2:1:0 ratio for interindividual variation: skin depth: AD status: temporal variation, respectively, although most of the community variation was associated with unmeasured parameters and stochastic processes (Fig. 5). Previously, interindividual variation of the skin bacteria has been linked to intrinsic (demography, age) and extrinsic factors (diet) (24), and among a plethora of other factors (25). Given this large source of variation, future studies should carefully consider the impact of the very high ratio of interindividual variation compared to AD. For example, high sampling numbers are required if more subtle findings regarding the skin-bacterial communities of AD are to be made.

A ‘core’ microbiome associated with skin has been previously hypothesized (26), and here we identified ‘core’ bacterial communities associated with individuals that were persistent over body locations, time, and between epidermal layers, accounting for approximately 25% of relative abundance and 20% (30 ASVs) of richness. While approximately 25% of these core ASVs were ubiquitously distributed, the remaining core community represents a sizeable quantity of ASVs that can be used to disentangle the bacterial communities of individuals over space and time.

In comparison to this interindividual variation, all other sources of variation analyzed within this study were relatively small. While the temporal variation of the skin microbiome has been more sparsely studied, temporal variation of the skin bacterial communities has mostly been found to be low within both healthy and AD skin (20, 26, 27). In both epidermal layers, we found negligible shifts in the bacterial communities over time. Flores et al. (27) found that the amount of variation of the bacterial communities varied substantially between healthy controls, and here we extend this finding to AD patients (i.e., patients varied between individuals), but there was no consistently higher/lower variation associated with AD skin. Additionally, even in the patients that exhibited the highest temporal variation, the majority of the more abundant ASVs were persistent throughout the sampling period. Together, results further support the hypothesis of a ‘microbial fingerprint’ (28), with highly individual communities that colonize individuals with and without AD, which remain distinguishable over periods of at least several months (29).

The bacterial communities at differing skin depths largely followed similar patterns of variation. Despite the communities within the epidermis having lower ASV richness, they did not better distinguish between AD patients and healthy controls (i.e., AD status explained similar amounts of community variation). Bacterial communities have been previously shown to differ between epidermal and dermal compartments (18), and between root hair follicles and the surface (3). Here, we demonstrate that there is a non-random spatial structure to the bacterial communities going through the epidermis (17) and that the inner skin bacterial communities are a subset of the outer (hence the reduced ASV richness within the epidermis) (18). Repeated tape stripping of the same location has previously been shown to remove 11.3% of the epidermal layer with five tapes (from a mean thickness of 56.9 μm to 50.4 μm) (22), suggesting that the bacterial communities vary at the micrometer scale within the longitudinal profile of the skin. Many ASVs were found nearly exclusively at the epidermal surface. These could be considered environmental contaminants, and repeated tape stripping of a single location on the body could therefore represent a less invasive method of sampling the ‘core’ patient microbiome without performing more invasive biopsies.

The effect of AD status on the bacterial communities is generally expected to be substantial (10). Counter to expectations, here we found only a small significant effect of AD on the community composition of both lesional and nonlesional skin from AD patients (Table 2), and no significant differences in α and β-diversity (Fig. S5). One potential reason for the limited effect of AD within this study was that patients here were presenting predominantly moderate AD (mean O-SCORAD = 20.5), which may have limited the effect size of skin condition on the bacterial communities. For example, Kong et al. (20) found that bacterial communities of nonlesional skin in children only significantly varied at the height of untreated flares (O-SCORAD = 42) compared to preflares (O-SCORAD = 22) and postflares (O-SCORAD = 18) as well as to healthy controls. Within our work, we found that differences in O-SCORAD were associated with shifts in the bacterial communities within both the lesional and nonlesional skin, thereby suggesting that the inclusion of patients with more severe AD might lead to larger differences being observed between the bacterial communities of healthy controls and AD patients (20, 30, 31). Additionally, the bacterial community composition significantly varied between AD patients with and without filaggrin mutations, both within lesional and nonlesional skin at both skin depths. Filaggrin mutations have similarly been found to differentiate the overall community composition of the bacteria within lesional and nonlesional skin from AD patients (32), with increased S. aureus abundance in the patients with mutations (33). Together, these results suggest that considering AD in presence-absence terms oversimplifies a dynamic system. Incorporating more complexity into future studies of AD might help unravel these potentially confounding variables to provide more clarity into how and why the skin bacteria differ in AD patients compared to healthy controls.

The bacterial communities of lesional skin significantly differed between different sampling locations, and to a much larger extent than other factors. Previously, body location has been found to have a major effect on determining the bacterial community (16, 18, 34), induced by differences in the skin microclimate (35). These differences between lesional communities may have therefore been driven in part by underlying differences between body locations. These results further typify how considering skin samples in discrete categorical groups (lesional, nonlesional, and healthy controls) overlooks complexity that ultimately may limit findings.

As expected, there was significantly higher Staphylococcus relative abundance (and richness at the surface only) associated with AD patients compared to healthy controls, but metabarcoding of the universal 16S rRNA region provides insufficient resolution to analyses Staphylococcus at the species level. Additionally, primer biases have been shown to systematically over/underrepresent taxa, and here we found a lack of Cutibacterium that are normally highly abundant within the skin bacterial communities. Bacterial primers targeting the universal V4 region of the bacterial rRNA have previously been characterized as underreporting Cutibacterium (36) and highlight another limitation of metabarcoding. Despite these limitations, several ASVs were identified that were much more persistent in AD patients than healthy controls (i.e., much more commonly found on AD patients), including many non-Staphylococcus species. While the majority of previous studies have focused on Staphylococcus species, others have also identified non-Staphyloccocus species that have been suggested to be common on AD patients' skin and are worthy of further exploration (14). However, when considering within-participant persistence, there were relatively few participants and a high number of tests, limiting the ability to find significance in identifying further ASVs associated with AD. Given decreasing sequencing costs, cohort studies can be performed on large numbers of participants with metagenome-assembled genomes to provide both high levels of confidence in the taxonomic assignments and functional annotations (37), which can ultimately be used to study the importance of non-Staphylococcus species within AD pathogenesis.

In this work, we stress the importance of considering the high levels of interindividual variation of the skin-associated bacteria, and that sampling subsurface level communities may effectively reduce ‘noise’ from bacterial environmental contaminants. However, we make several potential links to variation within AD patients on both the nonlesional and lesional skin, including AD severity and filaggrin mutations, while the lesional skin location on the body was further linked to variation in the lesional bacterial communities. Other recent studies have identified differences in the bacterial communities of AD patients, and considering AD as a presence/absence disorder is likely an oversimplification. Therefore, future studies could provide more insight into the role of the skin bacteria within AD pathogenesis by considering this further complexity of AD patients coupled with large sampling numbers. Ultimately, these could have implications for the management of the disorder, with optimal treatments tailored to the patient genome, immunological status, and microbiome composition.

## MATERIALS AND METHODS

### Participants.

Patients with AD were recruited from the outpatient clinic of the Department of Dermatology, Bispebjerg Hospital, from October 2017 to February 2018. A total of 20 AD patients over 18 years of age were recruited according to the UK criteria (38). Patients were excluded if they had treatment with phototherapy or systemic immunosuppressive drugs within 4 weeks of sampling. Most patients used intermittent treatment with topical corticosteroids (TCS) between visits and were instructed not to use TCSs on sample location 7 days before sampling. However, three patients had used TCS within 1 week before sampling. Additionally, over the same period, skin samples were obtained from 20 healthy volunteers, over 18 years of age and with no history of skin disorders. Participants ranged from 25 to 53 years old, with 14 men and 26 women sampled (a full list of patient metadata is in Data Set S1), with relatively even ages and sex ratios between healthy controls and AD patients. All participants were screened for filaggrin mutations within the R501X, 2282del4, and R2447X genes (33). The severity of AD was assessed using the objective scoring atopic dermatitis (O-SCORAD) (23). The study period lasted for 12 weeks, with sampling at 4-week intervals (Data Set S1). One AD patient was only sampled at the first two sampling time points, while another two AD patients had an O-SCORAD of zero on visits 3 and 4 (lesional samples were sampled in a previously lesional region). Oral antibiotic treatment within 4 weeks of study visit or between study visits had been used by three AD patients and two healthy controls, with one healthy control receiving oral antibiotics twice in the study period. Nonlesional skin samples from AD patients and healthy controls were taken from the volar forearm. However, lesional skin (LS) from AD patients were not always present on the volar forearm and were sampled across different body locations using the same approach and at the same time as the volar forearm was sampled.

The study was approved by the regional science ethics committee, Region H, Copenhagen, Denmark (protocol number H-16047983) and the Regional Danish Data Protection Agency (BFH-2017-042, I-Suite: 05449), and all participants provided signed informed consent.

### Sampling of stratum corneum.

At each sampling time point, the stratum corneum was collected using a tape-stripping technique (22, 39). Five consecutive adhesive, d-Squame tapes (22 mm diameter; CuDerm, Dallas TX, USA) were placed onto the skin and pressed for 10 s with standardized pressure (225 g/cm^2^, d-Squame Pressure Instrument D500). The tape strips were placed individually in cryo-vials and immediately stored at −80°C. The first (tape 1) and fifth (tape 5) tapes were processed for microbial analyses.

### DNA Extraction and sequencing preparation.

Tapes underwent DNA extraction using a DNeasy blood and tissue kit (Qiagen, Germany) per the manufacturer’s instructions, with a few modifications as previously outlined (39). Similarly, metabarcoding was performed targeting the universal V3-V4 16S rRNA region of bacteria using the 341F (5′-CCTAYGGGRBGCASCAG-3′) and 806R (5′-GGACTACNNGGGTATCTAAT-3′) primers. Sequencing preparation was carried out as fully described by Barnes et al. (39) using a TruSeq DNA PCR-Free Library Preparation Kits (Illumina, CA, USA). Completed libraries were sent for 250-paired end sequencing at the National High-Throughput Sequencing Center of Denmark (University of Copenhagen, Denmark) on an Illumina MiSeq platform (CA, USA).

### Bioinformatics.

Demultiplexed libraries underwent a second round of demultiplexing (using internal tags) using a custom script, with reads assigned to samples with only exact matches of the forward and reverse tags (available from http://github.com/tobiasgf/lulu). During this step, adapters, primers, and internal tags were also removed using CutAdapt (v1.9.1) (40) and reads of less than 400 bp removed using VSEARCH (v2.3.4) (41). A total of 42,322,973 reads were assigned to samples, accounting for a mean of 42,568.7 reads per sample. Reads from samples and extraction negatives will be made freely available at the Sequence Read Archive, the National Center for Biotechnology Information (NCBI) upon manuscript acceptance.

Demultiplexed reads were further processed using DADA2 (42) within the statistical computing environment R. Initially, demultiplexed reads were dereplicated and underwent quality filtering as error rates were calculated within DADA2. An initial sequence table was constructed before chimeras were identified using the removeBimeraDenovo function within DADA2. Reads from different runs were joined using mergeSequenceTables function.

Taxonomy was assigned using DADA2’s native naive RDP Bayesian classifier against the Silva 136 database (43). DADA2 produces ASVs, which unlike operational taxonomic units (OTUs) can differ by single base pairs. After processing, a total of 26,647,998 reads were assigned to samples, accounting for a mean of 42,568.7 reads per sample. ASVs were removed from samples if present in 10 or more of the negatives (roughly one-third of all negatives processed), removing 27 ASVs, including some of the most abundant ASVs within samples (a complete list of ASVs are in Table S1). Additionally, ASVs were removed if they occurred in less than 5 samples to further filter potential sequencing contaminants. Finally, reads not assigned to the bacteria or archaea, or assigned to mitochondria or chloroplast sequenced were also removed. Consequently, a mean of 12.7% of reads per sample was removed during these final processing steps. Read counts were normalized with a fourth-root transformation to counter PCR biases (44), which were then converted to relative abundances for downstream statistical analyses.

### Statistics.

All statistical analyses were performed in the statistical computing language *R* and visualized with ggplot2 package (45). For a comprehensive breakdown of every analysis, all R scripts are available (https://github.com/drcjbarnes/Temporal_and_spatial_variation_in_the_skin_bacteria.git). In this work, α-diversity was analyzed either as ASV richness or as Shannon’s diversity, which was calculated using the diversity function within the Vegan package (46). β-diversity was defined as the ASV difference between paired samples, community membership as Jaccard coefficients (proportion of shared ASVs), and community similarity as the Yue-Clayton similarity (weighted by relative abundance) with both calculated from custom scripts. Persistence was calculated as the proportion of samples an ASV was detected within, either across data sets or within participants, and was used as a measure of commonness for ASVs.

As part of initial analyses, only the healthy controls and nonlesional samples were processed. Community compositional effects were analyzed with PERMANOVA using the adonis2 function was used to analyze the community level variation associated with interindividual variation (patientID), tape depth, temporal variation, and AD status (nonlesional and healthy control). Similarly, these variables were correlated with ASV richness and Shannon’s diversity was performed using generalized linear modeling (GLM). Differences in β-diversity associated with interindividual variation and with AD status were calculated with Kruskal-Wallis tests.

Given the large differences in community richness and composition associated with the interindividual variation, all subsequent statistics were performed to incorporate this variation. Therefore, patientID was used as a grouping factor for all subsequent tests of community composition, and mixed linear modeling (MLM; using the LME4 package (47)) was performed for α- (ASV richness, Shannon’s) and β-diversity (ASV difference, community membership, and community similarity). This included comparing the AD communities at different skin depths (AD status to within/surface epidermal communities). Similarly, PERMANOVA and MLM were used to compare the lesional communities to the nonlesional and healthy control communities (separately) at each skin depth. Finally, PERMANOVA and MLM were used to investigate the community composition and diversity differences within the nonlesional and lesional groups at separate skin depths for AD-specific measures (as O-SCORAD and filaggrin mutations).

Significant variation in individual ASVs associated with different tape depths was identified on major ASVs (ASVs that occurred in more than 5 samples), using the within-participant persistence of each skin layer, which was performed with a Student’s *t* test. Results were corrected for multiple comparisons by converting *P* values to *Q* values via false discovery rate (FDR). The within-patient persistence was calculated for each skin depth separately (by aggregating sampling time points), and significant variation in the within-patient persistence (of individual ASVs) was assessed using mixed linear modeling. Patient ID was used as a random factor and AD status as a fixed effect, and *P* values were obtained using a correction for multiple comparisons and the false-discovery rate.

Significant differences in Staphylococcus abundance and ASV richness were calculated using mixed linear modeling.

### Data availability.

All Supplemental Material is freely available for download from the University of Copenhagen’s Electronic Research Data Archive (https://sid.erda.dk/sharelink/hkdDwYKYmc).
