# Erratum for Barnes et al., “Temporal and Spatial Variation of the Skin-Associated Bacteria From Healthy Participants and Atopic Dermatitis Patients”

**DOI:** 10.1128/msphere.00319-23

**Published:** 2023-09-28

**Authors:** Christopher J. Barnes, Maja-Lisa Clausen, Maria Asplund, Linett Rasmussen, Caroline Meyer Olesen, Yasemin Topal Yüksel, Paal Skytt Andersen, Thomas Litman, Anders Johannes Hansen, Tove Agner

## ERRATUM

Volume 7, issue 1, e00917-21, 2022, https://doi.org/10.1128/msphere.00917-21. Page 1: This article was published with Yasemin Topal Yüksel’s surname misspelled as “Yüsel” in the byline. The name should appear as given in this Erratum.

Page 12, “Bioinformatics” paragraph 1: The last sentence should read "Reads from samples and extraction negatives are freely available for download from the University of Copenhagen’s Electronic Research Data Archive (https://erda.ku.dk/archives/df76280733e748cac433f8ef28e558ab/published-archive.html)."

